# Prevalence of Helicobacter pylori Infection Among Rosacea and Chronic Spontaneous Urticaria Patients in a Tertiary Hospital in Riyadh, Saudi Arabia

**DOI:** 10.7759/cureus.17617

**Published:** 2021-08-31

**Authors:** Amal AlBalbeesi, Hanan Alsalman, Hend Alotaibi, Mona Halawani, Eman Almukhadeb, Fahad Alsaif, Nahla Azzam, Tuqa AlKaff, Mosfer Aldosari, Asem Shadid

**Affiliations:** 1 Department of Dermatology, College of Medicine King Saud University, Riyadh, SAU; 2 Gastroenterology, College of Medicine King Saud University, Riyadh, SAU; 3 Dermatology, King Fahad Medical City, Riyadh, SAU

**Keywords:** helicobacter pylori, rosacea, urticaria, urea breath test, innate immunity

## Abstract

Background: The multifactorial nature of rosacea and chronic spontaneous urticaria (CSU) pathogenesis complicates the achievement of satisfactory treatment outcomes. 13C urea breath test (UBT) has been identified as an accurate, non-invasive, and quick procedure to detect the presence of *Helicobacter pylori* (*H. pylori*) with high sensitivity and specificity.

Objective: In this study, we aim to assess the correlation between *H. pylori* infection and rosacea and CSU patients.

Methods: A cross-sectional, observational study was conducted on patients with rosacea and CSU in the dermatology clinic at King Khalid University Hospital in Riyadh, Saudi Arabia. History and physical examination were performed by a dermatologist. *H. pylori *13C-UBT detection was performed in all subjects.

Results: In total, 114 patients were included in this current study, with 41 rosacea and 73 urticaria patients. The vast majority of our subjects were females (96.5%). The mean (±SD) age was 42.3 (±12.7). More than half (58.8%) of the examined samples were positive for 13C-UBT; however, positive results were significantly higher in the rosacea patients (73.2%) compared to the urticaria group (50.7%), with a p-value of 0.019.

Conclusion: Our findings underline the significant association of *H. pylori* with rosacea and CSU regardless of the presence or absence of gastrointestinal symptoms. We thus recommend the inclusion of *H. pylori* testing in the routine workup of CSU and rosacea patients.

## Introduction

Rosacea has been defined as a chronic inflammatory relapsing disease of unknown etiology affecting mostly middle-aged adults, especially females more than males [1]. Rosacea commonly affects the central part of the face and is characterized by persistent or recurrent episodes of erythema, papules, pustules, and telangiectasias [2]. According to the American National Rosacea Society Expert Committee, there are four subtypes of rosacea, i.e., erythematotelangiectatic rosacea (ETR), papulopustular rosacea (PPR), phymatous rosacea, and ocular rosacea [3,4]. The pathogenesis of this disease remains to be unclear; however, it has been suggested that it may be the result of multifactorial chronic inflammation that is dominated by innate immunity as well as abnormal vasomotor function [5]. A recent systematic review and meta-epidemiological study found that rosacea affects approximately 5.46% of the adult population worldwide [6].

Chronic spontaneous urticaria (CSU), also referred to as chronic idiopathic urticaria, is a common and potentially debilitating skin condition whose primary characteristics involve the development of wheals and itching for a period of at least six weeks [7]. CSU is also defined as the occurrence of chronic urticaria with no clear cause, which, in fact, constitutes up to 70% of all cases [7,8]. CSU affects up to 1% of the general population, with variable duration of the disease that typically lasts for several months, but it may occasionally persist for decades [9]. Furthermore, this condition can affect both children and adults; it starts in the third to fifth decades of life and affects females almost twice as often as males [10,11]. Although there is no established theory that explains CSU pathogenesis [12], Philpott et al. showed that almost 30% of the CSU patients may have functional autoantibodies [13]. In fact, current evidence supports this finding, associating CSU with some common chronic infections, including *Helicobacter pylori* (*H. pylori*) [14,15].

*H. pylori* is a widely prevalent microbe that infects almost 50% of the world population and more than 80% of people living in developing countries like Saudi Arabia [16,17]. Data from epidemiological and experimental studies indicate a strong relation between *H. pylori* infection and the development of many extra gastric diseases, including several allergic and autoimmune diseases [18]. More specifically, previous studies have found an etiopathogenetic association between *H. pylori *infection and CSU in addition to possible skin improvements following its eradication [19,20]. In contrast, results from other studies have shown that *H. pylori *prevalence in afflicted patients does not differ from that of control groups [21,22]. Therefore, the association between *H. pylori* infection and CSU is still considered controversial.

Current evidence suggests that *H. pylori* is correlated to the development of rosacea [23,24]. However, this correlation remains to be debatable in literature. The prevalence of *H. pylori* infection among patients with rosacea has been assessed in many published reports [25-29], with the respective findings ranging from a prevalence of 100% to no significant differences between healthy people and rosacea patients [30,31].

Therefore, and while considering this controversy regarding the association between *H. pylori* infection and rosacea and CSU, this study aimed to assess this potential correlation among the population in Saudi Arabia. This study is of greater significance considering the scarcity of existing data on this subject in Saudi Arabia.

## Materials and methods

This cross-sectional observational study was conducted on patients with rosacea and CSU presented in the dermatology clinic at King Khalid University Hospital in Riyadh, Saudi Arabia from November 2015 to August 2019.

Patients’ demographic data, gastrointestinal and systemic symptoms, duration of symptoms, previous diagnosis of *H. pylori* or peptic ulcer disease, and the presence of the family history of *H. pylori* were recorded. Physical examination was performed by a qualified dermatologist in order to assess and record the clinical characteristics of both rosacea and CSU.

CSU severity was assessed based on the number of wheals per day and was divided into less than 20 wheals, 21-50 wheals, and more than 50 wheals. Meanwhile, itching was scored by the patients as either none, mild, moderate, or severe. Daily symptoms and the presence of symptom-free periods and dermatographism were recorded. For rosacea duration, the clinical subtype was determined based on the National Rosacea Society Expert Committee classification, which classifies rosacea into four subtypes, i.e., erythematotelangiectatic rosacea, papulopustular rosacea, phymatous rosacea, and ocular rosacea. Finally, severity was divided into mild, moderate, and severe [3,4].

*H. pylori* 13C-urea breath test (UBT) was obtained from all subjects enrolled. Patients were then asked to fast for 10 hours before the procedure. Breath was sampled at baseline before the consumption of 13C-urea-containing capsule and then again 30 minutes post-capsule consumption. The breath sample was analyzed for the detection of non-radioactive carbon-13-labeled isotopes with IRIS (infrared isotope analyzer); positive detection suggested the presence of *H. pylori*.

Inclusion criteria in this study involved adults from 18 to 60 years of age with either rosacea or CSU diagnosed by a dermatologist. In contrast, exclusion criteria included patients younger than 18 years and older than 60 years. Furthermore, patients with physical urticaria only and patients with known helminth infections or hepatitis B or C were excluded. Any patients who had recently been treated for *H. pylori*, or who were on proton pump inhibitors, or who received any antibiotics within the last six weeks were also excluded.

For statistical analysis, data were collected, managed, and coded in a spreadsheet using Microsoft Excel 2010® (Microsoft Corporation, New Mexico, USA). Data were analyzed using the Statistical Package for the Social Sciences (SPSS)® version 21.0 (IBM Inc., Chicago, Illinois, USA).

Descriptive analysis was performed, with categorical variables presented in the form of frequencies and percentages. In contrast, data exploration using the Shapiro-Wilk test was performed for the continuous variables; data were found to have a normal distribution and hence were presented as mean ± SD [range]. Consequently, independent t-tests were used to compare any two groups for continuous variables and the chi-squared test to compare proportions among the groups. A point-biserial correlation was used to measure the strength and direction of the association that existed between a continuous and a dichotomous variable. Moreover, we used a binary logistic regression to predict the probability that observation would fall into one of two categories of a dichotomous dependent variable based on one or more independent variables that were either continuous or categorical. Any output with a p below 0.05 was interpreted as an indicator of statistical significance. Informed consent was then obtained from all patients included in this study.

## Results

In total, 114 patients were included in this study, of which 41 patients had rosacea and 73 patients had chronic spontaneous urticaria. The vast majority of patients were females and of Saudi origin at 96.5% and 91.2%, respectively, with no statistically significant (p > 0.05) difference between the rosacea and urticaria groups. The mean (±SD) age of the participants was 42.3 (±12.7), which was non-significantly higher among the rosacea patients at 44.9 (±9.1) vs. 40.7 (±14.2) years in the CSU group, with a p-value of 0.093, as shown in Table 1.

**Table 1:** Demographic data *Statistically significant at 5% level of significance. UBT, Urea breath test.

Characteristic	Rosacea (n = 41) n (%)	Urticaria (n = 73) n (%)	P value	Total (n = 114) n (%)
Gender				
Male	1 (2.4)	3 (4.1)	0.642	4 (3.5)
Female	40 (97.6)	70 (95.9)		110 (96.5)
Age in years; Mean ± SD (Range)	44.9 ± 9.1 (23-59)	40.7 ± 14.2 (17-71)	0.093	42.3 ± 12.7 (17-71)
Nationality				
Saudi	38 (92.7)	66 (90.4)	0.681	104 (91.2)
Non-Saudi	3 (7.3)	7 (9.6)		10 (8.8)
UBT test (n = 114)				
Positive	30 (73.2)	37 (50.7)	0.019*	67 (58.8)
Negative	11 (26.8)	36 (49.3)		47 (41.2)
P value	<0.001*	0.866		0.007*
Duration of gastrointestinal symptoms in years; Mean ± SD (Range)	6.8 ± 5.9 (2 months-20.0)	5.7 ± 4.8 (2 months-20.0)	0.340	6.1 ± 5.2 (2 months-20.0)
Skin type				
2	0 (0.0)	1 (1.4)	0.449	1 (0.9)
3	8 (18.2)	7 (9.5)	0.182	15 (13.2)
4	18 (40.9)	52 (70.3)	0.002*	70 (61.4)
5	12 (27.3)	9 (12.2)	0.043*	21 (18.4)
6	3 (6.8)	4 (5.4)	0.762	7 (6.1)

With regard to the prevalence of *H. pylori*, more than half (58.8%) of the examined population was positive for 13C-UBT, with the positive percentage significantly higher in the rosacea patients at 73.2% compared to 50.7% in the urticaria group, with a p-value of 0.019. No significant difference was noted between the rosacea and CSU patients in terms of the mean duration of the disease. Skin type 4 was the most prevalent skin type in our population sample, representing 61% of the studied sample. This skin type was significantly higher among urticaria patients at 70.3% compared to 40.9% among rosacea patients as shown in Table 1.

The comparison between rosacea and CSU patients with respect to systemic and gastrointestinal symptoms is shown in Table 2. A significantly higher percentage of rosacea patients experienced weight loss compared to urticaria patients at 63.6% vs. 36.4%, respectively, with a p-value of 0.044. In contrast, a significantly higher percentage of CSU patients experienced less bloating compared to rosacea patients with a p-value of 0.005.

**Table 2:** Characteristics of urticaria (n = 73) *Statistically significant at 5% level of significance. UBT, Urea breath test.

Characteristic	Total (n = 73) n (%)	UBT Positive (n = 37) n (%)	UBT Negative (n = 36) n (%)	P value
Wheals/24H				
A: none	2 (2.7)	0 (0.0)	2 (100)	<0.001*
B: <20/24H	53 (72.6)	28 (52.8)	25 (47.2)	0.635
C: 21-50/24H	14 (19.2)	7 (50.0)	7 (50.0)	0.998
D: >50/24H	4 (5.5)	2 (50.0)	2 (50.0)	0.998
Itching				
Mild	22 (30.1)	11 (50.0)	11 (50.0)	0.998
Moderate	27 (37.0)	15 (55.6)	12 (44.4)	0.342
Severe	24 (32.9)	11 (45.8)	13 (54.2)	0.476
Symptoms almost daily				
Yes	32 (43.8)	18 (56.3)	14 (43.8)	0.401
No	41 (56.2)	19 (46.3)	22 (53.7)	
Symptom-free period				
Yes	54 (74.0)	28 (51.9)	26 (48.1)	0.737
No	19 (26.0)	9 (47.4)	10 (52.6)	
Dermatographism				
Yes	45 (61.6)	24 (53.3)	21 (46.7)	0.534
No	28 (38.4)	13 (46.4)	15 (53.6)	0.541

Furthermore, Table 3 presents the comparison between 13C-UBT-positive and 13C-UBT-negative patients. Dysphagia was higher among the 13C-UBT-positive group, but there were no statistically significant differences between the two groups in terms of other assessed parameters, including weight loss, bloating, abdominal gases, early satiety, heartburn, odynophagia, having no symptoms suggestive of* H. pylori*, and previous and personal or family history of *H. pylori*, since all p-values were > 0.05. Rosacea patients were more likely to have a positive 13C-UBT compared to urticaria patients, with a statistically significant difference. A statistical significance was noted in the prevalence of dysphagia, which was considered a main feature in the majority of the 13C-UBT-positive patients at 90.9% as opposed to only 9.1% of the 13C-UBT-negative patients.

**Table 3:** Comparing other characteristics with UBT test results (n = 144) *Statistically significant at 5% level of significance UBT, Urea breath test.

Characteristic	UBT Positive (n = 67) n (%)	UBT Negative (n = 47) n (%)	P value
Disease			
Rosacea (n = 41)	30 (73.2)	11 (26.8)	0.019*
Urticaria (n = 73)	37 (50.7)	36 (49.3)	
Weight loss			
Yes (n = 11)	6 (54.5)	5 (45.5)	0.764
No (n = 103)	61 (59.2)	42 (40.8)	
Bloating			
Yes (n = 58)	35 (60.3)	23 (39.7)	0.728
No (n = 56)	32 (57.1)	24 (42.9)	
Abdominal gases			
Yes (n = 68)	38 (55.9)	30 (44.1)	0.446
No (n = 46)	29 (63.0)	17 (37.0)	
Early satiety			
Yes (n = 24)	13 (54.2)	11 (45.8)	0.606
No (n=90)	54 (60.0)	36 (40.0)	
Heartburn			
Yes (n=68)	41 (60.3)	27 (39.7)	0.688
No (n = 46)	26 (56.5)	20 (43.5)	
Dysphagia			
Yes (n = 11)	10 (90.9)	1 (9.1)	0.025*
No (n = 103)	57 (55.3)	46 (44.7)	
Odynophagia			
Yes (n = 1)	0 (0.0)	1 (100)	0.412
No (n = 113)	67 (59.3)	46 (40.7)	
Symptoms			
Yes (n = 93)	56 (60.2)	37 (39.8)	0.510
No (n = 21)	11 (52.4)	10 (47.6)	
Previous diagnosis of *H. pylori*			
Yes (n = 28)	19 (67.9)	9 (32.1)	0.261
No (n = 86)	48 (55.8)	38 (44.2)	
Family history of *H. pylori*			
Yes (n = 37)	22 (59.5)	15 (40.5)	0.918
No (n = 77)	45 (58.4)	32 (41.6)	

Table 4 presents the characteristics of the urticaria patients in response to the 13C-UBT results. As per our findings, 100% of patients with no wheals showed negative 13C-UBT results, with a significant p-value of <0.001. However, there were no significant differences between the 13C-UBT-positive and 13C-UBT-negative patients in regards to itching severity, symptoms, symptom-free period, and dermatographism.

**Table 4:** Comparing other characteristics between rosacea and urticaria (n = 144) *Statistically significant at 5% level of significance. UBT, Urea breath test.

Characteristic	Rosacea (n = 41; 36%) n (%)	Urticaria (n = 73; 64%) n (%)	P value
UBT			
Positive (n = 67)	30 (44.8)	37 (55.2)	0.019*
Negative (n = 47)	11 (23.4)	36 (76.6)	
Weight loss			
Yes (n = 11)	7 (63.6)	4 (36.4)	0.044*
No (n = 103)	34 (33.0)	69 (67.0)	
Bloating			
Yes (n = 58)	28 (48.3)	30 (51.7)	0.005*
No (n = 56)	13 (23.2)	43 (76.8)	
Abdominal gases			
Yes (n = 68)	28 (41.2)	40 (58.8)	0.159
No (n = 46)	13 (28.3)	33 (71.7)	
Early satiety			
Yes (n = 24)	10 (41.7)	14 (58.3)	0.512
No (n = 90)	31 (34.4)	59 (65.6)	
Heartburn			
Yes (n = 68)	25 (36.8)	43 (63.2)	0.829
No (n = 46)	16 (34.8)	30 (65.2)	
Dysphagia			
Yes (n = 11)	4 (36.4)	7 (63.6)	0.977
No (n = 103)	37 (35.9)	66 (64.1)	
Odynophagia			
Yes (n = 1)	1 (100)	0 (0.0)	0.18
No (n = 113)	40 (35.4)	73 (64.6)	
Previous diagnosis of *H. pylori*			
Yes (n = 28)	8 (28.6)	20 (71.4)	0.348
No (n = 86)	33 (38.4)	53 (61.6)	
Family history of* H. pylori*			
Yes (n = 37)	15 (40.5)	22 (59.5)	0.48
No (n = 77)	26 (33.8)	51 (66.2)	

ETR has been identified as the most prevalent subtype of rosacea among our patients, with 85.4%. Both ETR and PPR were significantly associated with *H. pylori* with p-values of 0.014 and <0.001, respectively, thus showing that PPR was slightly more significant than ETR. The mean duration of rosacea did not differ significantly between the 13C-UBT-positive and 13C-UBT-negative patients with 6.5 (±6.4) and 8.0 (±8.1) years, respectively. Mild and moderate cases of rosacea were found to be significantly higher among the 13C-UBT-positive patients compared to 13C-UBT-negative patients (78.9% and 70% vs. 21.1% and 30%, respectively, with p-values of <0.001 and 0.023 as shown in Table 5).

**Table 5:** Types and other characteristics of rosacea (n = 41) *Statistically significant at 5% level of significance. UBT, Urea breath test.

Characteristic	Total (n = 41) n (%)	UBT Positive (n = 30) n (%)	UBT Negative (n = 11) n (%)	P value
Type of rosacea (More than 41 due to more than one type)				
Erythematotelangiectatic	35 (85.4)	25 (71.4)	10 (28.6)	0.014*
Papulo-pustular	12 (27.3)	10 (83.3)	2 (16.7)	<0.001*
Ocular	8 (18.2)	5 (62.5)	3 (37.5)	0.158
Other–Rhinophyma & Morbihan	1 (2.3)	1 (100)	0 (0.0)	<0.001*
Duration in years; Mean ± SD (Range)	6.8 ± 6.8 (9–25 months)	6.5 ± 6.4 (9-20 months)	8.0 ± 8.1 (1-25 months)	0.548
Severity				
Mild	19 (46.3)	15 (78.9)	4 (21.1)	<0.001*
Moderate	20 (48.8)	14 (70.0)	6 (30.0)	0.023*
Severe	2 (4.9)	1 (50.0)	1 (50.0)	0.998

## Discussion

Rosacea and CSU are encountered on a regular basis in the dermatology practice. The multifactorial nature of their pathogenesis, which remains unknown to a certain extent, contributes to the complexity of achieving satisfactory treatment outcomes. 13C-UBT is an accurate, non-invasive, and quick procedure for detecting the presence of *H. pylori* with high sensitivity (97%) and specificity (98%) [32]. We conducted this study aiming to assess the correlation between *H. pylori* infection and rosacea and CSU.

We found that *H. pylori* was prevalent in more than half of the examined sample and was significantly higher in patients with rosacea than with CSU. Several studies have been published on the prevalence of *H. pylori* infection among patients with rosacea [25-29,33]. This prevalence ranges from 100% in some studies to other studies indicating that there is no significant difference between the rosacea patients and healthy people [31]. The estimated prevalence of rosacea in this current study is considered higher (73.2%) compared to the findings of McColl et al. (65.4% in 26 patients aged 26-82 years) [16,25]. In contrast, a higher prevalence (88%) was reported in another study [34]. Jørgensen et al. [35], in their systematic review and meta-analysis study, have reported that studies using the 13C-UBT for the diagnosis of *H. pylori* infection are more likely to identify a stronger association between rosacea and *H. pylori* compared to studies using serological tests [36], something which is also evident in our results.

Immunological and inflammatory factors could explain the association of *H. pylori* with rosacea. It is postulated that skin changes caused by *H. pylori* are induced through two distinct mechanisms. First, *H. pylori* are known to increase the production of nitrous oxide vasodilation and inflammation. The second mechanism is by the expression of cytotoxic genes A (cytotoxin-associated gene A, cagA), TNF-α, and IL-8, which subsequently initiate an inflammatory cascade of reactions. Furthermore, along with complement activation, the toxic factor of *H. pylori *can induce skin alterations resulting in the known clinical presentation of rosacea [37].

A previous study has shown that 25% of the CSU patients were positive for *H. pylori*, which is approximately half of the prevalence estimated in our study (50.7%) [37]. In addition, a lower prevalence of 16.5% was also reported in another study that included 266 chronic urticaria patients [37,38]. However, the high prevalence of *H. pylori* infection in patients diagnosed with CSU in this study is consistent with a previous meta-analysis that showed the prevalence of *H. pylori* infection was higher among CSU patients [38].

Various theories have been suggested in an attempt to explain the association between *H. pylori* infection and CSU. For instance, *H. pylori* infection increases the permeability of the gastric lining and therefore increases the risk of exposure to allergens in the gastrointestinal tract. In addition, antibodies against *H. pylori *could stimulate the release of histamine in the skin, thus promoting the formation of urticarial lesions [39,40].

The difference in the prevalence of *H. pylori* in rosacea and CSU across different published studies could be explained by several factors, such as different rates of *H. pylori *prevalence among different countries and the varying statistical and evaluation methods used in different studies. Also, research studies have used different strains with variable virulence, a fact that may help explain the discrepancy in association between *H. pylori* and rosacea/CSU [41].

Overall, our study showed a female preponderance, which is consistent with the current literature [1,42], where it is well-known that rosacea is more common among females. This might also explain the hormonal role in the pathogenesis of these gram-negative bacteria [43,44]. Furthermore, chronic urticaria exhibits a significant female preponderance, with an average female to male ratio of approximately 2-4:1 [45]. Some authors suggest that the low levels of dehydroepiandrosterone (DHEA)-S in females may attribute to the hormonal role in CSU [46]. However, definitive conclusions cannot be reached as current data remain scarce to support such a relationship. An additional hypothesis that could also explain this issue is that females are more likely to be exposed to different allergens and triggers as a result of daily exposure to household dust and handling of raw food for food preparation, thus making them more vulnerable to *H. pylori* infection, which, in turn, could trigger CSU [47]. Finally, another reason is that females are more likely to seek medical help compared to males [48].

Several studies have demonstrated higher rates of *H. pylori* infection among PPR patients and lower rates among ETR patients [1,24,42]. However, in our study, both PPR and ETR subtypes were significantly associated with *H. pylori* infection.

Saleh et al. [49] have compared *H. pylori* antigen-negative and positive subjects and found a statistically greater number of severe rosacea in patients with *H. pylori*-positive results than with *H. pylori*-negative ones. In addition, the number of mild cases of rosacea in the *H. pylori*-negative group was determined to be statistically higher compared to the cases of severe rosacea in the *H. pylori*-positive group. In our case, the prevalence of mild and moderate rosacea cases was significantly higher among *H. pylori*-positive patients. This indicates a tendency for rosacea to progress among *H. pylori*-positive rosacea patients.

The limitation of our study is the lack of control subjects as well as that we did not study the progression of cutaneous symptoms after initiation of the anti-helicobacter pylori treatment protocol.

## Conclusions

In conclusion, higher rates of *H. pylori *infection have been detected in patients with rosacea and CSU. Rosacea patients were found to be more likely to have positive 13C-UBT compared to CSU patients. Both ETR and PPR are closely related to *H. pylori* infection, with PPR being slightly more significant. There were no significant differences in the clinical characteristic between 13C-UBT-positive and negative patients among CSU patients.

## Appendices

Data-sharing statement

- The data that support the findings of this study are available from the corresponding author upon reasonable request.
